# Nitrite modulates aminoglycoside tolerance by inhibiting cytochrome heme-copper oxidase in bacteria

**DOI:** 10.1038/s42003-020-0991-4

**Published:** 2020-05-27

**Authors:** Yongting Zhang, Kailun Guo, Qiu Meng, Haichun Gao

**Affiliations:** 1 Institute of Microbiology College of Life Sciences, Zhejiang University, Hangzhou, 310058 Zhejiang China; 2 College of Biotechnology and Bioengineering, Zhejiang University of Technology, 18 Chaowang Road, Hangzhou, 310014 Zhejiang China

**Keywords:** Antibiotics, Bacteriology

## Abstract

As a bacteriostatic agent, nitrite has been used in food preservation for centuries. When used in combination with antibiotics, nitrite is reported to work either cooperatively or antagonistically. However, the mechanism underlying these effects remains largely unknown. Here we show that nitrite mediates tolerance to aminoglycosides in both Gram-negative and Gram-positive bacteria, but has little interaction with other types of antibiotics. Nitrite directly and mainly inhibits cytochrome heme-copper oxidases (HCOs), and by doing so, the membrane potential is compromised, blocking uptake of aminoglycosides. In contrast, reduced respiration (oxygen consumption rate) resulting from nitrite inhibition is not critical for aminoglycoside tolerance. While our data indicate that nitrite is a promising antimicrobial agent targeting HCOs, cautions should be taken when used with other antibiotics, aminoglycosides in particular.

## Abstract (web summary)

Zhang et al. show that nitrite mediates bacterial tolerance to aminoglycosides without affecting other antibiotics. They find that nitrite inhibits cytochrome heme-copper oxidases, blocking bacterial uptake of aminoglycosides. This study suggests nitrite as a promising antimicrobial agent while discouraging its combined use with aminoglycosides.

## Introduction

Nitrite, a ubiquitous nitrogen species, is the central player in the nitrogen biogeochemical cycle, linking nitrate to gas nitrogen or ammonium^1^. During its reduction to nitrogen, nitrite can be converted to nitric oxide (NO), a reactive free radical that interferes with protein cofactors, such as Fe–S clusters, heme, and lipoamide, or promotes the formation of reactive nitrogen species^2–4^. Although it has been widely accepted that antibacterial effects of nitrite, as used in food preservation for centuries, are attributable to NO formation^5^, many studies in recent years have revealed that nitrite has NO-independent effects^6–8^.

Nitrite (nebulized sodium nitrite) is currently in a phase 2b clinical trial as a drug for pulmonary hypertension^9^. The study has demonstrated that nitrite could be safely applied to reach millimolar concentrations in the airway surface liquid, paving the road for the agent to be a previously unidentified antimicrobial therapy. Nitrite has also been found to be either cooperative or antagonistic when used in combination with antibiotics in *Pseudomonas aeruginosa*^8,10,11^. These effects are a result of the bacteriostatic action, which does not require NO as an intermediate^1^. Antagonistic effects are commonly associated with aminoglycosides and also observed with ciprofloxacin (Cip), one of the quinolones that interfere with bacterial DNA gyrase^10,12^. In contrast, cooperative antimicrobial activities have been observed when used with polymyxins, polycationic lipopeptide antibiotics that interact with negatively charged lipopolysaccharide on the cell surface of Gram-negative bacteria^8^.

Tolerance induced by nitrite to aminoglycosides and Cip is proposed to be attributed to impaired respiration (oxygen consumption rate, hereafter referred to as respiration) given similar effects of other respiratory inhibitors, such as cyanide and carbonyl cyanide *m*-chlorophenylhydrazone (CCCP)^10,11^. As nitrite is bacteriostatic, this proposal is in perfect agreement with the recent report that antibiotic efficacy is linked to bacterial cellular respiration^13^. However, given that aminoglycosides have long been proposed to depend on proton motive force (PMF) for uptake^14^, it is possible that nitrite-mediated aminoglycoside tolerance may not be a direct result of reduced respiration. Unfortunately, in most, if not all, of these previous studies^10–13^, respiration is virtually an equivalent of PMF, because the observations were from the proton gradient inhibitors, with CCCP being the most commonly used one. Thus, although reduced respiration likely compromises PMF formation, the direct evidence is lacking. More importantly, despite these findings, the antimicrobial mechanisms of nitrite, especially those through which respiration and/or PMF are impaired, is only partially understood^11^.

Given that dependence on PMF for uptake of aminoglycosides is commonly found in both Gram-negative and Gram-positive bacteria, we hypothesize that nitrite must inhibit proteins that are well conserved. Moreover, we also hypothesize that the targets are likely to be out of the cytoplasm, because nitrite as a charged molecule could not be efficiently imported in at least some of these bacteria. By this logic, we focus on the respiratory chain, especially terminal oxidases. This coincides with the findings of systematic investigations into nitrite and NO physiology in *Shewanella oneidensis*, a facultative Gram-negative γ-proteobacterium renowned for respiratory versatility^15–20^. In this bacterium, the primary target of nitrite is heme-copper oxidase (HCO) *cbb*_3_ (hereafter referred to as *cbb*_3_), whereas NO indistinguishably interacts with all cytochromes *c*^15,16,19,20^.

The bacterial respiratory chain is generally branched after the quinone pool, linking to multiple terminal oxidases (oxygen reductases) that carry out reduction of O_2_ into H_2_O and simultaneously form PMF^21^. Prokaryotic terminal oxidases are characterized into two major groups: HCOs and the *bd*-type quinol oxidases^22^. Representative HCOs include cytochrome *aa*_3_ oxidases (mitochondrial-like oxidases, hereafter referred to as *aa*_3_) as in *Bacillus subtilis* and *Staphylococcus aureus*, cytochrome *bo*_3_ oxidases (hereafter referred to as *bo*_3_) as in *Escherichia coli*, and *cbb*_3_ as in *S. oneidensis* and *P. aeruginosa*.

In this study, we tested our hypotheses that targets of nitrite are conserved proteins in the respiration chain whose inhibition underlies increased tolerance to aminoglycosides with *E. coli*, *S. oneidensis*, *P. aeruginosa*. *B. subtilis*, and *S. aureus*. We found that although nitrite inhibits killing of aminoglycosides in all of these bacteria, the efficacy varies from one species to another. Effects of nitrite on individual HCOs were then assessed in the same genetic background and results revealed that *cbb*_3_ is much sensitive than *bo*_3_ and *aa*_3_. We further provided evidence to suggest that nitrite blocks the activity of aminoglycosides but not of other antibiotics and this action is dependent on PMF rather than impaired respiration. Overall, these data suggest that nitrite is a promising antimicrobial agent targeting HCOs, but cautions should be taken when used with other antibiotics, aminoglycosides in particular.

## Results

### Nitrite induces bacterial tolerance to aminoglycosides

**Fig. 1:**
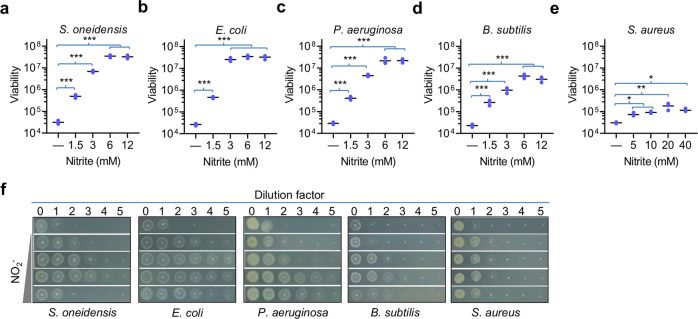
Nitrite modulates Gent susceptibility of various bacteria. **a**–**e** Time-kill analysis of Gent (5× MIC) and nitrite combination for *S. oneidensis*, *E. coli*, *P. aeruginosa*, *B. subtilis*, and *S. aureus*. For all strains, cultures at the mid-exponential phase were used and their cell numbers before the treatment were as follows: *S. oneidensis*, ∼2 × 10^8^ CFU/ml; *E. coli*, ∼2 × 10^8^ CFU/ml; *P. aeruginosa*, ∼2 × 10^8^ CFU/ml; *B. subtilis*, ∼5 × 10^7^ CFU/ml, and *S. aureus*, ∼2 × 10^8^ CFU/ml. Values shown were the number of viable cells 4 h after the treatment began. Impacts of nitrite on these bacteria were assessed and presented in Supplementary Fig. S1. Combination therapy was compared against monotherapy with Gent, between which statistically significant difference caused by nitrite were given (*n* ≥ 3, *P < 0.05; **P < 0.01; ***P < 0.001). **f** The spot dilution assay of Gent (5× MIC) and nitrite combination for indicated bacteria. Cultures prepared to contain ~10^9^ CFU/ml were regarded as the undiluted (dilution factor, 0), which were subjected to tenfold series dilution. Five microliters of each dilution was dropped on LB agar plates containing Gent and nitrite, whose concentrations were the same as shown in **a**–**f**. Results were recorded after incubation of 18 h. Experiments were performed independently at least three times and representative data were presented.

Nitrite has been found to block the activity of aminoglycosides in *P. aeruginosa*^10^. To test whether this phenomenon is common among bacteria, in this study we examined impacts of nitrite on aminoglycoside tolerance in *S. oneidensis*, *E. coli*, *B. subtilis*, and *S. aureus*, along with *P. aeruginosa* as the control. Susceptibility of all test strains to nitrite was first evaluated with the spot dilution test, to determine the proper concentration range for the study (Supplementary Fig. 1). *S. oneidensis* was much more sensitive to nitrite than *E. coli* and *S. aureus* as reported before^19^, whereas *P. aeruginosa* and *B. subtilis* showed nitrite tolerance in between. Time-kill kinetics of these strains were assessed by using the same approach for *E. coli*, *P. aeruginosa*, and *S. aureus* described previously^10,13^. Cultures for each strain grown aerobically in lysogeny broth (LB) to the mid-exponential phase (∼0.4 of OD_600_) were exposed to gentamicin (Gent) or streptomycin (Str) at 5× minimum inhibitory concentration (MIC) in the absence and presence of nitrite. For Gram-negative bacteria under test, the addition of nitrite 1.5–12 mM induced elevated tolerance to Gent (Fig. 1a–c). Although the induction appeared to be dose-dependent with nitrate at low concentrations (<3 mM), saturating effects were observed when higher concentrations were applied. The effects of nitrite on tolerance of Gram-positive bacteria, *B. subtilis* and *S. aureus*, to Gent, albeit visible only at much higher concentrations, were also dose-dependent (Fig. 1d–e). Apparently, nitrite was much less effective on these two bacteria, especially *S. aureus*, when compared with the three Gram-negative counterparts. Similar results were obtained from Str (Supplementary Fig. 2), suggesting that the phenomenon is likely common to aminoglycosides.

For confirmation, we performed the spot dilution test. The droplets of exponential-phase cultures containing different numbers of cells were applied onto LB agar plates supplemented with Gent and nitrite. Clearly, nitrite was very effective in inducing tolerance of the three Gram-negative bacteria to Gent, up to 4-log increase in the number of viable cells (Fig. 1f). On the contrary, the ability of nitrite to modulate Gent tolerance of two Gram-positive bacteria was relatively weak. Although nitrite at the most effective concentration improved viability of *B. subtilis* cells by up to 2-log, it rescued cell survival <1-log in *S. aureus*. The contrast between Gram-negative and Gram-positive bacteria regarding the effect of nitrite on modulation of aminoglycoside tolerance suggests that physiological differences matter.

We then made attempts to determine whether the observed effects of nitrite are via NO. As this has been comprehensively addressed in the case of *P. aeruginosa*^10^, here we only repeated time-kill assays in the presence of PTIO (2-Phenyl-4,4,5,5-tetramethylimidazoline-1-oxyl-3-oxide), an NO scavenger. With all bacteria tested, results obtained in the presence of excessive PTIO were comparable to those in its absence (Supplementary Fig. 3). Moreover, the differences in data from the experiments without and with PTIO were statistically insignificant. As *S. oneidensis* could not produce NO^19^, it served as a perfect control for eliminating impacts of excessive PTIO on physiology. These data were in perfect agreement with the previous finding that nitrite induces tolerance to aminoglycoside antibiotics without involving NO.

### Nitrite modulates aminoglycosides tolerance by inhibiting *cbb*_3_

**Fig. 2:**
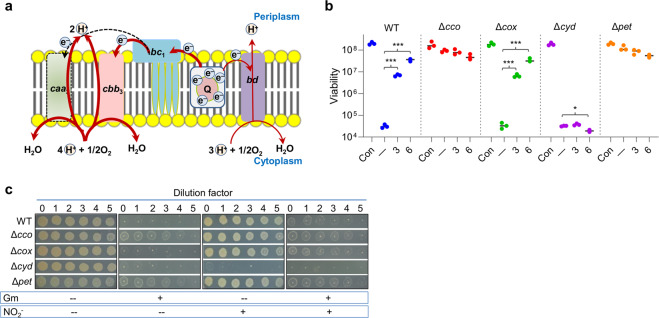
*cbb*_3_-based respiration is essential in nitrite-mediated Gent tolerance. **a** Model illustrating oxygen respiration in *S. oneidensis*. *S. oneidensis* possesses two HCOs, *cbb*_3_, and *caa*_3_, which obtain electrons from the quinol pool via the *bc*_1_ complex, and a quinol oxidase, *bd*. For oxygen respiration, *cbb*_3_ dictates and *bd* is its subordinate, whereas activity of *caa*_3_ is not detected under normal conditions. **b** Time-kill analysis of Gent (5× MIC) and nitrite (given in mM; −, no nitrite) combination for relevant *S. oneidensis* mutants. Con, no addition of Gent and nitrite. WT, wild type; Δ*cco*, *cbb*_3_-less; Δ*cox*, *caa*_3_-less; Δ*cyd*, *bd*-less; Δ*pet*, *bc*_1_-less. Asterisks indicate statistically significant difference between values being compared (*n* ≥ 3, *P < 0.05; **P < 0.01; ***P < 0.001). **c** The spot dilution assay of Gent (5× MIC) and nitrite (3 mM) combination for indicated bacteria. Experiments were performed independently at least three times and representative data were presented.

It is well known that the uptake of aminoglycoside antibiotics by bacterial cells is an energy-requiring process that depends on PMF^14^. The *S. oneidensis* genome encodes three terminal oxidases, HCOs *caa*_3_ and *cbb*_3_, as well as non-HCO *bd*, among which *cbb*_3_ and *bd* oxidases are produced and exhibit activity under normal conditions^16^ (Fig. 2a). For oxygen respiration, *cbb*_3_ is the predominant oxidase, whereas *bd* functions as an accessory oxidase and, more importantly, supports aerobic growth in the absence of *cbb*_3_^16^. Given that *cbb*_3_ is proposed to be the primary target of nitrite in *S. oneidensis*^15^, we hypothesized that nitrite modulates aminoglycoside tolerance by inhibiting terminal oxidases.

To test this, we examined Gent susceptibility of the *cbb*_3_-deficient strain (Δ*cco*) constructed, verified, and characterized previously, which loses cytochrome *c* oxidase activity completely^16^. Time-kill assays revealed that the *cbb*_3_ loss induced tolerance to Gent substantially, more effective than the addition of nitrite at any concentrations (Fig. 2b). A comparable effect was observed from a mutant lacking the cytochrome *bc*_1_ complex (Δ*pet*) (Fig. 2b), through which *cbb*_3_ obtains electrons from the quinone pool (Fig. 2a), supporting that the induction of Gent tolerance is attributable to the deficiency in *cbb*_3_-based respiration. In contrast, the deletion of *cox* (encoding *caa*_3_) genes (Δ*cox*) did not elicit any detectable difference in Gent tolerance without or with nitrite when compared with the wild type, which is in perfect agreement with the fact that the enzyme is not produced to physiological relevant levels^16,23^. In the case of *bd*, its absence (Δ*cyd*) did not affect Gent tolerance without nitrite (Fig. 2b), suggesting that *bd* activity is not critically implicated in Gent tolerance. However, it should be noted that the effect of nitrite on Gent tolerance could not be assessed directly, because the depletion of *bd* vastly sensitized *S. oneidensis* cells^15,24^.

We then made attempts to verify these observations with the spot dilution test. The loss of *cbb*_3_, albeit only modestly, reduces aerobic growth rates, whereas neither *caa*_3_ nor *bd* affects growth when *cbb*_3_ is present^16^. Consistent results were obtained from LB agar plates (Fig. 2c), although the growth difference between the wild-type and Δ*cco* (the same for Δ*pet*) strains appeared even smaller. Clearly, the *cco* and *pet* mutants exhibited substantially elevated Gent tolerance, whereas the strains lacking either *caa*_3_ or *bd* behaved as the wild type. Importantly, the Δ*cyd* strain was hypersensitive to nitrite, while all other strains under test had comparable nitrite susceptibility. This hypersensitivity prevented Δ*cyd* cells from growing on LB agar plates containing 3 mM nitrite, a condition that blocked Gent activity in the wild-type and Δ*cox* strains. These data, all together, manifest that nitrite modulates aminoglycoside susceptibility by compromising *cbb*_3_-based respiration, implying that *cbb*_3_, the *bc*_1_ complex, or both could be targets of nitrite. This study focused on *cbb*_3_, because it has been shown to be susceptible to nitrite^15–20^.

### HCOs are susceptible to nitrite

Given that other HCOs, including *caa*_3_, *aa*_3_, and *bo*_3_, are capable of proton-pumping during oxygen respiration as *cbb*_3_, we reasoned that they modulate aminoglycoside susceptibility through a similar mechanism. To test this, genes encoding *caa*_3_ (*S. oneidensis cox* operon), *bo*_3_ (*E. coli cyo* operon), and *aa*_3_ (*S. aureus qox* operon), and the required accessary proteins were expressed in the *S. oneidensis* wild-type and Δ*cco* strains (Supplementary Fig. 4a), and their influences on aminoglycoside susceptibility without or with nitrite in *S. oneidensis* were examined. By this way, all HCOs are compared in the same genetic background to avoid interference of physiological differences of bacteria from which each HCO comes (Fig. 1).

**Fig. 3:**
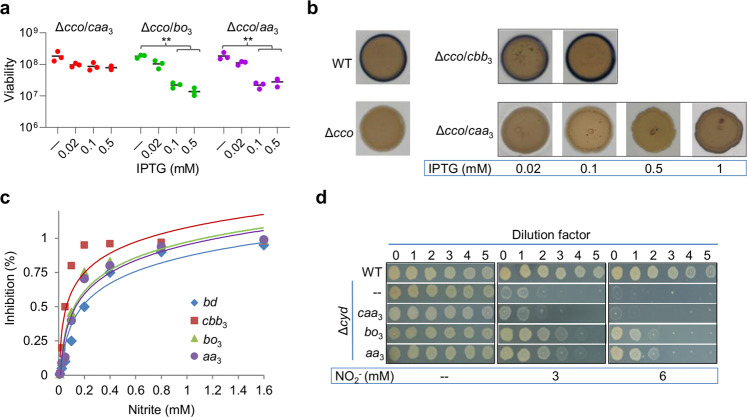
HCOs are susceptible to nitrite. HCOs, including *S. oneidensis caa*_3_, *E. coli bo*_3_, and *S. aureus aa*_3_, were expressed in the *S. oneidensis* Δ*cco* strain as described in Supplementary Fig. S4A. Expression was driven by IPTG-inducible promoter P*tac*. **a** HCOs in excess inhibit growth of *S. oneidensis*. Shown were cell densities (by CFU) of the Δ*cco* strain expressing one of HCOs with IPTG at indicated concentrations, which were derived from 6 h cultures presented in Supplementary Fig. S4B. Asterisks indicate statistically significant difference between values being compared (*n* ≥ 3, *P < 0.05; **P < 0.01; ***P < 0.001). **b** Activity of *cbb*_3_ and *caa*_3_ revealed by the Nadi assay. The method is based on the rapid formation of indophenol blue from colorless α-naphtol catalyzed by cytochrome *c* oxidase, using DMPD as an exogenous electron donor. Results shown were photographed 2 min after the reaction started. ∆*cco/cbb*_3_ represented the genetically complemented strain, in which a copy of *cco* was driven by P*tac*. **c** Nitrite susceptibility of HCOs. Respiration rates of membranes containing one of the indicated oxidases prepared from cells grown with 0.1 mM IPTG were measured as a function of nitrite concentrations. Lines represent single exponential fits to the data (Table 1) for IC_50_ values. **d** Nitrite susceptibility of the *S. oneidensis* Δ*cyd* strain expressing one of HCOs with 0.1 mM IPTG. Experiments were performed independently at least three times and representative data were presented.

When isopropyl β-d-1-thiogalactoside (IPTG) was supplemented up to 1 mM, the expression of *S. oneidensis caa*_3_ did not significantly affect growth of either the wild-type or Δ*cco* strain (Fig. 3a and Supplementary Fig. 4b). In contrast, *E. coli bo*_3_ and *S. aureus aa*_3_ produced with 0.1 mM IPTG substantially inhibited growth. We then determined whether these proteins have oxidase activity in *S. oneidensis*. The Nadi assay was employed for assessing *caa*_3_ activity, because the approach specifically detects cytochrome *c* oxidase-dependent activity^25^. Using *N*, *N*-dimethyl-p-phenylenediamine monohydrochloride as an exogenous electron donor, cytochrome *c* oxidase catalyzes the rapid formation of indophenol blue from colorless *a*-naphthol. As reported before^16^, the Δ*cco* strain expressing *caa*_3_ with IPTG up to 0.5 mM barely showed cytochrome *c* oxidase activity, contrasting the wild-type and the Δ*cco* strains expressing *cco* with 0.02 mM IPTG (Fig. 3b). However, when overproduced with 1 mM IPTG, *caa*_3_ did exhibit residual activity.

To determine the activity of *E. coli bo*_3_ and *S. aureus aa*_3_ produced in *S. oneidensis*, membrane preparations of the Δ*cco*Δ*cyd* strain expressing each of HCOs were used to measure oxygen respiration in a cell-free amperometric assay^3,26^. It should be noted that no aerobic growth was observed from the Δ*cco*Δ*cyd* strain expressing *caa*_3_, even with 1 mM IPTG, whereas both *E. coli bo*_3_ and *S. aureus aa*_3_ supported growth, albeit slowly (Supplementary Fig. 4c). Oxygen consumption was examined with the membranes (∼200 μg/ml) using 5 mM NADH as the electron donor. Respiratory activities of the membranes of *S. oneidensis* cells expressing *E. coli bo*_3_ in the presence of 0.02 and 0.1 mM IPTG were recorded as ~256 and 564 μM O_2_/mg protein/s, respectively (Table 1). From *S. oneidensis* cells expressing *S. aureus aa*_3_ in the presence of 0.02 and 0.1 mM IPTG, activities were recorded as ∼481 and 814 μM O_2_/mg protein/s, respectively. To compare the relative sensitivities to nitrite inhibition, we measured the half-maximal inhibitory concentration for nitrite (IC_50_(NO_2_^−^)) for both *E. coli bo*_3_ and *S. aureus aa*_3_ in the *S. oneidensis* membranes (Table 1). At ∼70 μM O_2_, the IC_50_(NO_2_^−^) values for *E. coli bo*_3_ and *S. aureus aa*_3_ were ∼120 ± 13 and 137 ± 15 μM, respectively (Fig. 3c). These values are substantially higher than that for *S. oneidensis cbb*_3_ (57 ± 6 μM) but still lower than that for *bd* (207 ± 18 μM). We then examined nitrite susceptibility of *S. oneidensis* Δ*cyd* strains expressing *E. coli bo*_3_ and *S. aureus aa*_3_. Indeed, these foreign oxidases, when expressed with 0.1 mM IPTG, elevated nitrite tolerance, albeit still more sensitive to nitrite than the wild type (Fig. 3d). All of these data indicate that bacterial HCOs are generally substantially more sensitive to nitrite than *bd*.

**Table 1:** Respiration activity of membranes carrying different oxidases^a^.

Strain	Oxidases	IPTG (mM)	Respiration activity (μmol mg^−1^ s^−1^)	IC_50_(NO_2_^−^) (μM)
Δ*cyd*	*cbb*_3_	—	248 ± 36	57 ± 6
Δ*cco*	*bd*	—	58 ± 6	207 ± 18
Δ*cco*Δ*cyd*/*bo*_3_	*bo*_3_	0.02	256 ± 31	120 ± 13
		0.1	564 ± 64	124 ± 15
Δ*cco*Δ*cyd*/*aa*_3_	*aa*_3_	0.02	481 ± 59	137 ± 15
		0.1	814 ± 72	140 ± 21

^a^Enzyme activities (μmol O_2_/mg protein/s) were measured in the presence of 5 mM NADH. Values are means ± SDs of at least three independent experiments.

### HCOs regulate nitrite-mediated aminoglycoside susceptibility

**Fig. 4:**
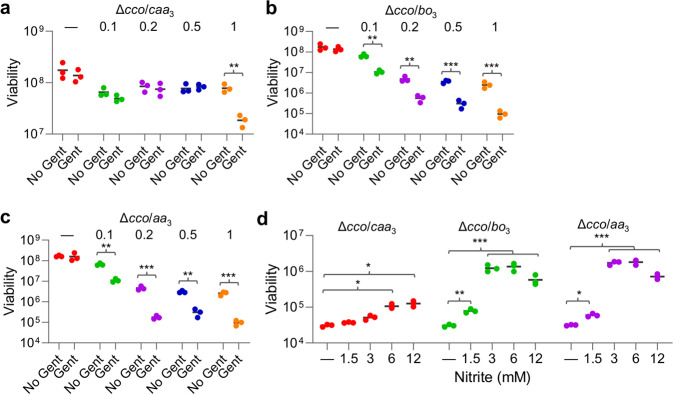
HCOs regulate nitrite-mediated aminoglycoside susceptibility. Cell numbers before the treatment were ∼2 × 10^8^ CFU/ml. Time-kill analysis of Gent (5× MIC) for the Δ*cco* strain expressing one of HCOs with IPTG at indicated concentrations. **a** *caa*_3_, **b** *bo*_3_, and **c** *aa*_3_. Asterisks indicate statistically significant difference between values being compared (*n* ≥ 3, *P < 0.05; **P < 0.01; ***P < 0.001). **d** Time-kill analysis of Gent (5× MIC) and nitrite combination for the Δ*cco* strain expressing one of HCOs. IPTG for *caa*_3_, 1 mM; for *bo*_3_ and *aa*_3_, 0.1 mM. Asterisks indicate statistically significant difference between values being compared (*n* ≥ 3, *P < 0.05; **P < 0.01; ***P < 0.001).

To assess the roles of *caa*_3_, *bo*_3_, and *aa*_3_ in nitrite-mediated aminoglycoside susceptibility, we examined Gent susceptibility of the Δ*cco* strain expressing one of these oxidases. In the presence of 1 mM IPTG, *caa*_3_ could elicit a slight decrease in Gent tolerance but failed to do so when IPTG was supplemented at 0.5 mM or lower (Fig. 4a). In the presence of 0.02 mM IPTG, the Δ*cco* strain expressing either *E. coli bo*_3_ or *S. aureus aa*_3_ became more sensitive to Gent (Fig. 4b, c). Similarly, these two oxidases produced with 0.1 mM or above IPTG sensitized cells, although in the meantime they impaired growth. We then examined the effect of nitrite on Gent tolerance of the Δ*cco* strain expressing one of these oxidases (IPTG concentrations for *caa*_3_, 1 mM, and for *bo*_3_ and *aa*_3_, 0.1 mM). Expressions of *E. coli bo*_3_ and *S. aureus aa*_3_ substantially decreased Gent tolerance of the Δ*cco* strain, even with 1.5 mM nitrite (Fig. 4d). Expectedly, *S. oneidensis caa*_3_ exhibited extremely low effectiveness: statistically significant differences were observed only from nitrite at 6 and 12 mM. All of these data suggest that these oxidases play a role in terms with aminoglycoside susceptibility similar to that of *cbb*_3_ in *S. oneidensis*.

### PMF but not respiration dictates uptake of aminoglycosides

**Fig. 5:**
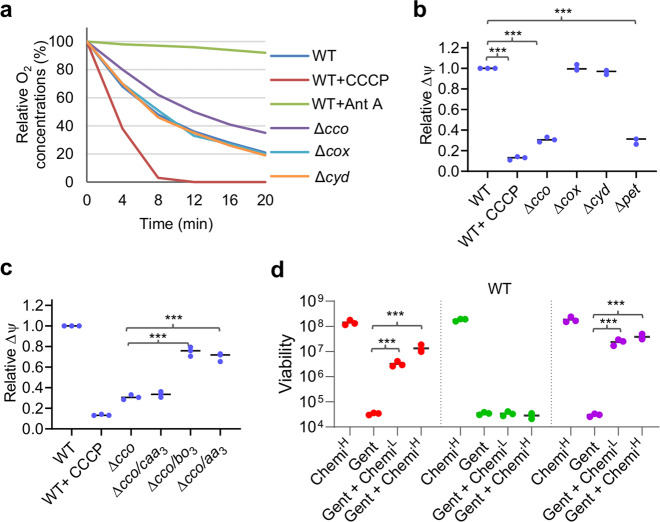
PMF but not respiration dictates uptake of aminoglycosides. **a** Oxygen consumption in whole cells. Oxygen consumption was measured with a cell density of 10^8^ cells/ml immediately after the addition of indicated chemicals. Antimycin A (Ant A) and CCCP were added at final concentrations of 10 μg/ml and 50 μM, respectively. AU, arbitrary units. **b** Impact of oxidases and exposure to ionophores on membrane potentials. Measurement of fluorescence intensity using dye DiOC_2_. The averaged fluorescence intensity of the mutants was normalized to that of the wild type, which was set to 1, giving relative Δψ. Statistics values were deduced on the basis of comparisons with the wild type. CCCP, 50 μM. Asterisks indicate statistically significant difference between values with asterisks and that of the wild type (*n* ≥ 3, ***P < 0.001). **c** Impact of oxidases and exposure to ionophores on membrane potentials. Asterisks indicate statistically significant difference between values being compared (*n* ≥ 3, *P < 0.05; **P < 0.01; ***P < 0.001). **d** Time-kill analysis of Gent (5× MIC) and KCN (red), BDQ (green) or CCCP (purple) combination for WT (∼2 × 10^8^ CFU/ml before the treatment). Chemi, one of the chemicals with superscript L and H representing low and high concentrations (100 and 500 μM for KCN, 1 and 5 μM for BDQ, 10 and 50 μM for CCCP, respectively). Asterisks indicate statistically significant difference between values being compared (*n* ≥ 3, *P < 0.05; **P < 0.01; ***P < 0.001). Experiments were performed independently at least three times and data were presented as means ± SEM.

It is well known that the uptake of aminoglycoside antibiotics by bacterial cells is an energy-requiring process that depends on PMF^14^. However, recent lines of evidence have suggested a link between antibiotic efficacy and bacterial cellular respiration^13^. Thus, it is possible that nitrite induces tolerance to aminoglycosides by impairing bacterial cellular respiration. To test this, we measured oxygen consumption rates of strains lacking each of three oxidases. As shown in Fig. 5a, the wild-type, Δ*cox*, and Δ*cyd* strains exhibited indistinguishable oxygen consumption and CCCP expectedly stimulated oxygen consumption substantially. In contrast, the Δ*cco* strain was modestly defective, in comparison with the effect of electron transport inhibitor antimycin A, which abolished oxygen consumption (Fig. 5a). Thus, the *cbb*_3_ loss only mildly affects oxygen consumption rate, in line with the finding that it slightly impairs aerobic growth^16^. We then directly measured the generation of the membrane potential (ΔΨ) of these strains. After de-energization of the membrane potential by starvation, cells incubated in LB were stained with DiOC_2_ and fluorescence intensities were assayed. The wild-type cells were clearly energized but became depolarized by the addition of protonophore CCCP (Fig. 5b). Compared with the wild type, both the Δ*cco* and Δ*pet* strains demonstrated a dramatic loss of ΔΨ, whereas the Δ*cox* and Δ*cyd* strains displayed residual depolarization, signifying maintenance of a ΔΨ (Fig. 5b). Moreover, impacts of *caa*_3_, *bo*_3_, and *aa*_3_ on ΔΨ were assessed in the Δ*cco* strain and results demonstrated that *bo*_3_ and *aa*_3_ elevated ΔΨ of Δ*cco* cells, whereas *caa*_3_ was ineffective (Fig. 5c).

To further support that PMF is the primary factor for aminoglycoside tolerance. Respiration inhibitor KCN and respiration stimulator bedaquiline (BDQ), which target HCOs and the ATPase, respectively^27^, as well as PMF destroyer CCCP, were compared in terms of their impacts on Gent tolerance of the *S. oneidensis* wild-type and Δ*cco* strains. Treatments of the wild-type strain with KCN and CCCP resulted in substantially increased tolerance to Gent, whereas no significant effect was observed from BDQ (Fig. 5d). In contrast, the Δ*cco* strain was not responsive to any of these drugs (Supplementary Fig. 5). As BDQ differs from the other two inhibitors in that it stimulates respiration without significantly altering ΔΨ^28^, these data support that PMF rather than respiration efficacy dictates the uptake of aminoglycoside antibiotics.

### HCOs are not involved in tolerance to other antibiotics

**Fig. 6:**
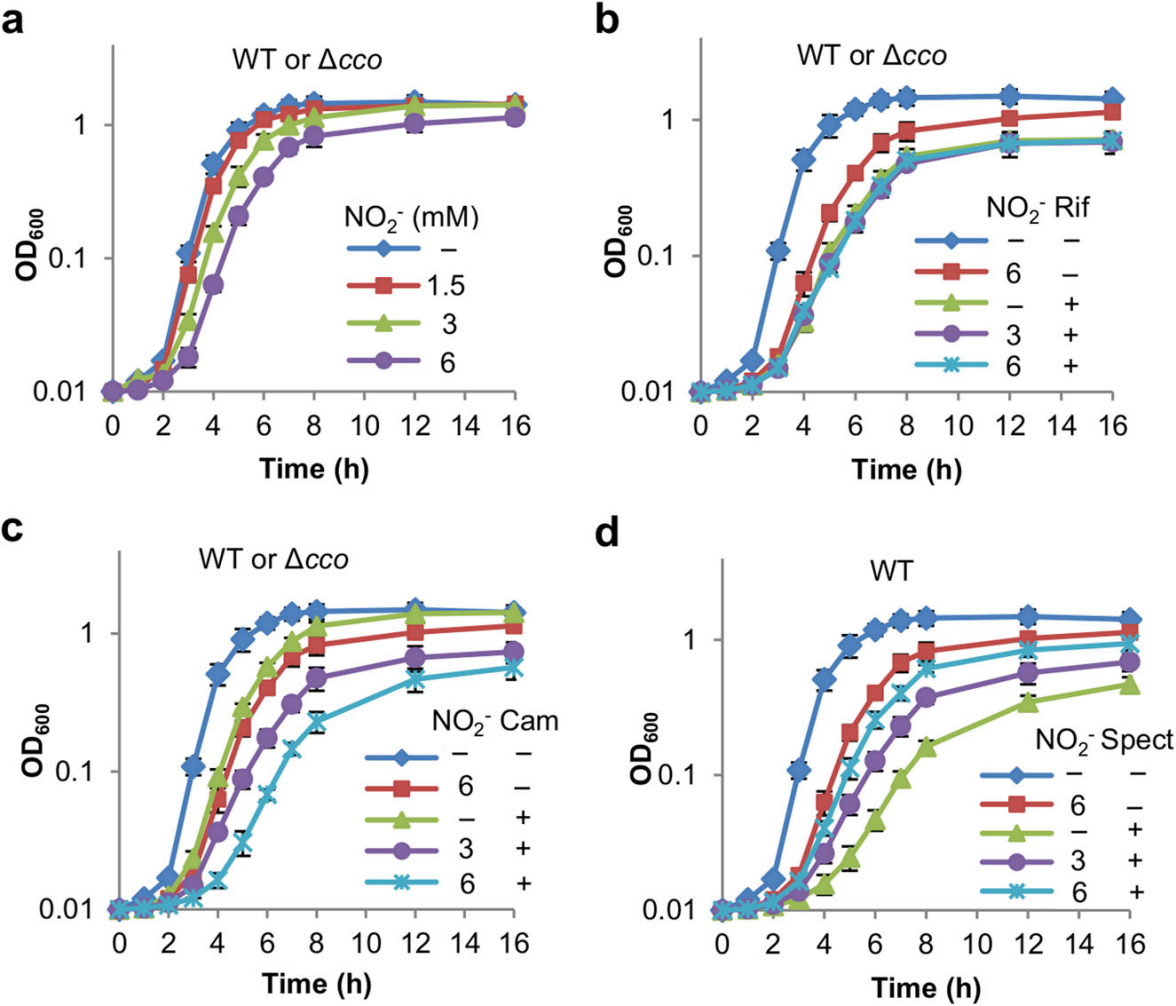
HCOs are dispensable in modulating susceptibility to antibiotics other than aminoglycosides. **a**–**d** Growth analysis of antibiotics (0.2× MIC) and nitrite combination for relevant *S. oneidensis* strains in liquid LB. Growth was recorded by measuring OD_600_ values of cultures. Experiments were performed independently at least three times and data were presented as means ± SD.

We further tested whether nitrite also mediates susceptibility of the *S. oneidensis* wild-type and Δ*cco* strains to chloramphenicol (Cam, bacteriostatic) and rifampin (Rif, bactericidal), whose targets are in the cytoplasm as aminoglycosides. In addition, polymyxin and Cip, with which nitrite shows cooperative and antagonistic activity, respectively, against *P. aeruginosa* biofilms, were also included^8,11^. The time-kill assay revealed that nitrite had little effect on the killing of Rif, polymyxin, or Cip, regardless of the presence of *cbb*_3_ or not (Supplementary Fig. 6a). Similar results were observed with Cam (Supplementary Fig. 6a), which is expected, because this assay is not effective for bacteriostatic antibiotics. We then compared the influence of nitrite (3 and 6 mM) on growth of these strains in the presence of these antibiotics at the sublethal concentrations. Clearly, nitrite inhibited growth of the wild-type and Δ*cco* strains comparably, and this inhibition was independent of Rif (Fig. 6a, b). In contrast, nitrite aggravated the inhibitory effect of Cam on growth of both strains, implicating that their impacts are additive (Fig. 6c). For further verification, we assessed growth of these two strains with nitrite and spectinomycin (Spect), a bacteriostatic aminoglycoside. Expectedly, nitrite improved growth of the wild type in the presence of Spect (1 μg/ml) (Fig. 6d). Importantly, this effect was not evident for the Δ*cco* strain (Supplementary Fig. 6b), further supporting that HCOs are essential for nitrite-mediated uptake of aminoglycosides.

## Discussion

Nitrite has emerged as an attractive pharmaceutical molecule in recent years, although it has been used as a bacteriostatic agent in food preservation for centuries. Nebulized sodium nitrite, a hypoxia-sensitive NO-dependent selective pulmonary vasodilator^29^, has been advanced into randomized trials in pulmonary arterial hypertension (PAH) patients^9^. Although further efforts are required to demonstrate the efficacy of nebulized sodium nitrite in PAH patients, the data at the current stage provide sufficient evidence to support the safe use of inhaled nitrite in the clinical setting. This serves great motivation for attempts to use nitrite as an antimicrobial agent alone and in combination with other antibiotics, and not surprisingly, many have been made^6,8,10,11^.

There have been a myriad of investigations into the mechanisms by which nitrite inhibits growth of bacteria. The prevailing theory is that the process is NO-dependent: nitrite is converted to NO in cells, which in turn directly inhibits many cellular targets^5^. An overwhelming majority of the NO targets identified to date are cytoplasmic metabolic enzymes with essential redox-active centers that comprise of Fe–S clusters, hemes, or protein thiols^2–4^. In this study, we show that HCOs are the most vulnerable cellular target of nitrite in bacteria, as proposed before^15^. Despite this, the possibility that the *bc*_1_ complex is an equally critical target of nitrite could not be ruled out and effects to test this are underway. Growth inhibition of nitrite in vivo, however, appears very modest, if not undetectable, in many bacteria, because they are additionally equipped with non-HCO oxidases, such as *bd* and alternative oxidases (AOXs), which are highly resistant to nitrite and NO^3,15,24,30^.

The data presented here manifest that nitrite induces tolerance to aminoglycosides in both Gram-negative and Gram-positive bacteria. However, it is clear that nitrite is more effective on Gram-negative than on Gram-positive bacteria. Given that all bacterial HCOs are susceptible to nitrite, other physiological aspects may be accountable for the difference. Recent studies have linked to aminoglycoside susceptibility with various metabolites^31,32^. Although some metabolites potentiate aminoglycoside sensitivity in both Gram-negative and Gram-positive bacteria, others can enable aminoglycoside activity in Gram-negative (*E. coli*) but not Gram-positive bacteria (*S. aureus*)^31^. We thus speculate that that nitrite may impact distinct metabolic pathways and/or same metabolic pathways to varying extent, modulating PMF and thereby aminoglycoside susceptibility.

In addition to *cbb*_3_, three other HCOs that are widely found in bacteria, *caa*_3_, *bo*_3_, and *aa*_3_, were examined for their roles in nitrite-mediated tolerance to aminoglycosides. In *E. coli* and *S. aureus*, *bo*_3_ and *aa*_3_ are the primary oxidases for oxygen respiration, respectively. In line with this, expression of either of these two HCOs impacts *S. oneidensis* physiology. Both HCOs produced at proper levels support growth of strains lacking all native oxidases, largely restore ΔΨ of the *S. oneidensis cbb*_3_-less mutant, and sensitize the cells to aminoglycosides, in comparison with the wild type. When in excess, either *bo*_3_ or *aa*_3_ severely inhibits growth of the *S. oneidensis cbb*_3_-less mutant, a widely reported phenomenon whose underpinning mechanism remains unanswered^33^. Moreover, our data demonstrated that both *E. coli bo*_3_ and *S. aureus aa*_3_ are more tolerant to nitrite and their expression confers the *S. oneidensis bd*-less mutant increased tolerance to nitrite. Nevertheless, it is conceivable that these oxidases play a dispensable role in tolerance of their native hosts to nitrite given the presence of *bd*s and AOXs.

In the case of *caa*_3_, its physiological role in *S. oneidensis* and also in *P. aeruginosa* is negligible under normal conditions because of extremely low expression^16,33,34^. In this study, we detected cytochrome *c* oxidase activity from the *S. oneidensis cbb*_3_-less mutant expressing *caa*_3_ in the presence of 1 mM IPTG, supporting that the enzyme could function when expressed to physiologically relevant levels^23,35^. However, given that *S. oneidensis* and *P. aeruginosa* mutants that lack all other oxidases are unable to grow aerobically, even with *caa*_3_ forcibly produced at substantially elevated levels, it is possible that the oxidase may be only residually active, as suggested before^16,35^. Therefore, we were not surprised to find that the contribution of *S. oneidensis caa*_3_ to respiration, PMF generation, nitrite tolerance, or nitrite-mediated aminoglycoside tolerance is hardly detected even when overproduced.

Nowadays, it is quite common to use antibiotic combinations to treat bacterial infections. However, many in vitro studies have demonstrated attenuation of bactericidal activity across a range of drugs and organisms when used with a bacteriostatic agent^36–38^. It has been proposed that this is due to reduction of respiratory/metabolic rates by bacteriostatic agents, which lowers bactericidal antibiotic efficacy^13,39^. However, despite a bacteriostatic agent, nitrite does not modulate aminoglycoside tolerance mainly by impairing respiration/metabolism; instead, nitrite compromises PMF generation by directly inhibiting HCOs. We found that tolerance to aminoglycosides is not affected by BDQ, which stimulates respiration without significantly affecting ΔΨ^28^. Moreover, by expressing *cbb*_3_, *bo*_3_, and *aa*_3_ in the same genetic background, we were able to show that the efficacy of these enzymes in PMF generation is comparable. The correlation between the restoration of ΔΨ by *bo*_3_ and *aa*_3_ in the *cbb*_3_-less mutant and lowered tolerance to aminoglycosides supports that PMF is critical for uptake of aminoglycosides. Interestingly, with respect to respiration, non-HCO oxidases almost suffice, based on the observation that the HCO-deficient mutants grow only marginally slower than the wild type^15,16,20^. In contrast, non-HCO oxidases are inferior to HCOs in generating PMF, because they could not pump protons^21,22^. These differences therefore further support that PMF rather than respiration is critical for nitrite-modulated tolerance to aminoglycosides.

To date, there have been many reports regarding modulation of nitrite on bacterial tolerance to antibiotics, aminoglycosides in particular^8,11^. However, the effects observed are not consistent and even contradicting in some cases. Here we have carried out a comprehensive investigation into the mechanisms underlying nitrite-mediated aminoglycoside tolerance in multiple model bacteria. By comparing aminoglycoside susceptibility of strains lacking each of terminal oxidases and producing one of possible HCOs without and with nitrite, we discover that HCO-based respiration is essential to nitrite-mediated aminoglycoside tolerance. In contrast, HCOs are dispensable in modulating susceptibility to antibiotics other than aminoglycoside. Using biochemical inhibitors of the electron transport chain, we present evidence to show that nitrite-mediated aminoglycoside tolerance occurs through the generation of PMF rather than consumption of oxygen. Collectively, these data support nitrite as an antimicrobial agent targeting HCOs of bacteria but demand careful consideration of its application when used in combination with antibiotics.

## Methods

### Strains, plasmids, and culture conditions

*E. coli* and *S. oneidensis* strains and plasmids used in this study were listed in Table 2. In addition, *P. aeruginosa* strain PA14, *S. aureus* strain ATCC 25923, and *B. subtilis* strain 168 were used in this study. Sequences of the primers used in this study were available upon request. All chemicals are from Sigma-Aldrich, Co., unless otherwise noted. *E. coli* and *S. oneidensis* were grown aerobically in Lysogeny broth (LB, Difco, Detroit, MI) at 37 °C and 30 °C, respectively, for genetic manipulation. When appropriate, the growth medium was supplemented with the following: 2, 6-diaminopimelic acid (DAP), 0.3 mM; ampicillin (Sangon, Shanghai), 50 μg/ml; kanamycin, 50 μg/ml; and Gent, 15 μg/ml.

**Table 2:** Strains and plasmids used in this study.

Strain or plasmid	Description	Source/reference
*E. coli* strain		
MG1655	Wild type	ATCC 700926
WM3064	Donor strain for conjugation; Δ*dapA*	W. Metcalf, UIUC^a^
*S. oneidensis* strain		
MR-1	Wild type	ATCC 700550
HGCYD	Δ*cyd* derived from MR-1	^43^
HG2364-1	Δ*cco* derived from MR-1	^27^
HG0608-10	Δ*pet* derived from MR-1	^44^
HG2364-3285	Δ*cydB*Δ*ccoN* derived from MR-1	^16^
HG4606-9	Δ*cox* derived from MR-1	This study
Plasmid		
pHGM01	Ap^r^ Gm^r^ Cm^r^ suicide vector	^24^
pHGEN-P*tac*	IPTG-inducible P*tac* expression vector	^25^
pHGEN-P*tac*-*cox*	For inducible expression of *S. oneidensis caa*_3_	This study
pHGEN-P*tac*-*cyo*	For inducible expression of *E. coli bo*_3_	This study
pHGEN-P*tac*-*qox*	For inducible expression of *S. aureus aa*_3_	This study

^a^UIUC, University of Illinois Urbana-Champaign.

### Antibiotic tolerance assessment

To prepare samples for antibiotic tolerance assessment, overnight cultures were inoculated into fresh LB by 200× dilution, shaken at 200 r.p.m. at 30 °C (*S. oneidensis*) and 37 °C (*E. coli*, *P. aeruginosa*, *B. subtilis*, and *S. aureus*) on a rotating shaker at 250 r.p.m. in flasks, and growth was recorded by measuring optical density at 600 nm (OD_600_). Cells grown to the mid-exponential phase (∼0.4 of OD_600_, the same throughout the study) were collected and adjusted to proper concentrations for subsequent analyses.

Time-kill assays were performed as described elsewhere^13^. Cells were plated in a six-well dish and antibiotics were added at 5× MIC for all bacteria under test, unless otherwise noted: Gent 2.5 μg/ml, Str 5 μg/ml, Cip 1 μg/ml, Rif 1 μg/ml, and colistin (polymyxin) 2.5 μg/ml. To generate biological equivalents of cell killing, 5 μg/ml Gent and 10 μg/ml Str were used for *P. aeruginosa* and 5 μg/ml Gent for *S. aureus*. Aliquots of 200 μl were taken at specified times, serially diluted, and spot-plated onto LB agar plates, to determine colony-forming units per ml (CFU/mL). Dilutions that grew 50–200 colonies were counted. For the spot dilution test, on LB agar plates.

The spot dilution test was employed to evaluate viability and growth inhibition on plates essentially the same as described previously^40^. In brief, cells at the mid-exponential phase were collected by centrifugation and adjusted to 10^9^ cells/ml, which was set as undiluted (dilution factor 0). Tenfold serial dilutions were prepared with fresh LB medium and 5 μl of each dilution was dropped onto LB plates containing antibiotics MIC and/or nitrite, in the form of sodium nitrite (Sangon, Shanghai). The plates were incubated at 30 °C for 18 h or as indicated when different before being read. In addition, growth inhibition assays were used to evaluate impacts of bacteriostatic antibiotics at 0.2× MIC: Cam 2 μg/ml and Spect 8 μg/ml.

### In-frame mutant construction

In-frame deletion strains were constructed using the *att*-based fusion PCR method as described previously^41^. In brief, the fusion fragments were generated from the first round of PCR products flanking the genes of interest and introduced into pHGM01, and the resulting vectors were transformed into and maintained in *E. coli* DAP auxotroph WM3064. After verification, the correct vectors were transferred into relevant *S. oneidensis* strains via conjugation, allowing integration of the fusion constructs into the chromosome. Verified transconjugants were screened for gent-sensitive and sucrose-resistant colonies. Mutants were verified by sequencing the mutated region.

### Controlled gene expression

Plasmid pHGEN-P*tac* was used for genetic complementation of the mutants generated in this study^42^. Genes of interest were generated by PCR, cloned into the vector, and the resultant vectors were transformed into *E. coli* WM3064. After verification by sequencing, the vectors were transferred into the relevant *S. oneidensis* strains via conjugation. Expression of the cloned genes was controlled by IPTG (Abcam Shanghai)-inducible promoter P*tac*. For expressing *S. aureus aa*_3_, the *qoxABCD* operon was first cloned behind P*tac* and then the *ctaM* gene was placed after the operon but before the terminator sequence.

### Cytochrome oxidase activity assay

The Nadi assay was used for visual analysis of cytochrome *c* oxidase activity^26^. Five microliters of each culture at the mid-log phase under test was dropped onto LB plates and the plates were incubated for 24 h. A solution of 0.5% α-naphthol in 95% ethanol and 0.5% DMPD (N,N-dimethyl-ρ-phenylenediamine monohydrochloride) was applied to cover the droplets developed. Formation of indophenol blue was timed as an indicator of cytochrome *c* oxidase activity.

Solubilized membranes were prepared for quantitative analysis of the cytochrome oxidase activity as described previously^24^. In brief, cell pellets were resuspended in 20 mM Tris-HCl (pH 7.6) supplemented with DNase I and protease inhibitors, and disrupted by French Press. After debris and unbroken cells removing, the membranes were pelleted by ultracentrifugation for 1 h at 230,000 × *g* at 4 °C and subsequently resuspended in 20 mM Tris-HCl pH 7.6 with 5% glycerol to a protein concentration of 10 mg/ml. Solubilization was performed with *n*-dodecyl β-d-maltoside (DDM) to a final concentration of 1% (w/v) on a rotary tube mixer for 2 h at 4 °C. The DDM-solubilized membranes were obtained by collecting the supernatant after ultracentrifuging for 1 h at 230,000 × *g* at 4 °C. Pellets, containing 30–40 mg/ml of protein, were resuspended in sodium phosphate buffer pH 7.5, 50 mM NaCl, 10% glycerol, and the suspension was stored at −80 °C if not immediately used. The cytochrome oxidase activity was assayed as a measure of oxygen consumption rates using an OxyGraph oxygen electrode (Hansatech), using NADH as an electron donor according to the methods described previously^3^. The IC_50_ values of the cytochrome *bd* and *cbb*_3_-HCO for nitrite were obtained from plots of rates against nitrite concentrations.

The OxyGraph oxygen electrode (Hansatech) was also used to measure oxygen consumption rates of whole bacterial cells, according to the manufacturer’s instructions. *S. oneidensis* cells collected from cultures grown to the mid-exponential phase in LB by centrifugation were suspended to 1 × 10^8^ cells/ml in sterile distilled water and oxygen consumption was measured at 25 °C.

### Membrane potential measurements

Membrane potential (ΔΨ) measurements were carried out with the *Bac*Light™ Bacterial Membrane Potential Kit (ThermoFisher). Cells of bacteria under test were grown to the mid-exponential phase in LB were collected, washed and resuspended in PBS buffer for 2 h at 30 °C. After starvation, cells were adjusted to the same cell density (∼10^7^ cells/ml), incubated with 30 μM DiOC_2_ in LB at room temperature for 30 min in the dark. ΔΨ was analyzed using a FACSCalibur flow cytometer (Becton Dickinson) according to the manufacturer’s guidelines. As a depolarized control, the protonophore carbonyl cyanide *m*-chlorophenylhydrazone (CCCP) was added at a final concentration of 5 μM where indicated.

### Statistics and reproducibility

Each experiment was performed at least three times independently with identical experimental settings, to ensure reproducibility. The experiments were not randomized and the investigators were not blinded to bacteria strain genotypes during experiments. Sample sizes for each experiment are indicated in the figures and figure legends. Statistical tests were performed in Microsoft Excel and GraphPad Prism 7 software. Statistical significance was presented as follows: *p < 0.05, **p < 0.01, and ***p < 0.001.

### Reporting summary

Further information on experimental design is available in the Nature Research Reporting Summary linked to this paper.

## Supplementary information

Supplemental Information

Description of Additional Supplementary Files

Supplementary Data 1

Reporting Summary

## Data Availability

The data that support the findings of this study are included in the paper or available from the corresponding author upon request. The source data presented in the main figures are provided in Supplementary Data 1.

## References

[1] Maia LB, Moura JJG (2014). How biology handles nitrite. Chem. Rev..

[2] Hyduke DR, Jarboe LR, Tran LM, Chou KJY, Liao JC (2007). Integrated network analysis identifies nitric oxide response networks and dihydroxyacid dehydratase as a crucial target in *Escherichia coli*. Proc. Natl Acad. Sci. USA.

[3] Mason MG (2009). Cytochrome *bd* confers nitric oxide resistance to *Escherichia coli*. Nat. Chem. Biol..

[4] Richardson AR (2011). Multiple targets of nitric oxide in the tricarboxylic acid cycle of *Salmonella enterica* Serovar Typhimurium. Cell Host Microbe.

[5] Reddy D, Lancaster J, Cornforth D (1983). Nitrite inhibition of *Clostridium botulinum*: electron spin resonance detection of iron-nitric oxide complexes. Science.

[6] Major TA (2010). Sodium nitrite-mediated killing of the major cystic fibrosis pathogens *Pseudomonas aeruginosa*, *Staphylococcus aureus*, and *Burkholderia cepacia* under anaerobic planktonic and biofilm conditions. Antimicrob. Agents Chemother..

[7] Bueno M, Wang J, Mora AL, Gladwin MT (2013). Nitrite signaling in pulmonary hypertension: mechanisms of bioactivation, signaling, and therapeutics. Antioxid. Redox Signal..

[8] Zemke AC (2014). Nitrite modulates bacterial antibiotic susceptibility and biofilm formation in association with airway epithelial cells. Free Radic. Biol. Med..

[9] Rix PJ (2015). Pharmacokinetics, pharmacodynamics, safety, and tolerability of nebulized sodium nitrite (AIR001) following repeat-dose inhalation in healthy subjects. Clin. Pharmacokinet..

[10] Zemke AC, Gladwin MT, Bomberger JM (2015). Sodium nitrite blocks the activity of aminoglycosides against *Pseudomonas aeruginosa* biofilms. Antimicrob. Agents Chemother..

[11] Zemke AC, Kocak BR, Bomberger JM (2016). Sodium nitrite inhibits killing of *Pseudomonas aeruginosa* biofilms by ciprofloxacin. Antimicrob. Agents Chemother..

[12] Correia S, Poeta P, Hébraud M, Capelo JL, Igrejas G (2017). Mechanisms of quinolone action and resistance: where do we stand?. J. Med. Microbiol..

[13] Lobritz MA (2015). Antibiotic efficacy is linked to bacterial cellular respiration. Proc. Natl Acad. Sci. USA.

[14] Taber HW, Mueller JP, Miller PF, Arrow AS (1987). Bacterial uptake of aminoglycoside antibiotics. Microbiol. Rev..

[15] Fu H (2013). Crp-dependent cytochrome *bd* oxidase confers nitrite resistance to *Shewanella oneidensis*. Environ. Microbiol..

[16] Zhou G (2013). Combined effect of loss of the *caa*_3_ oxidase and Crp regulation drives *Shewanella* to thrive in redox-stratified environments. ISME J..

[17] Jin M, Fu H, Yin J, Yuan J, Gao H (2016). Molecular underpinnings of nitrite effect on cyma-dependent respiration in *Shewanella oneidensis*. Front. Microbiol..

[18] Jin M, Zhang Q, Sun Y, Gao H (2016). NapB in excess inhibits growth of *Shewanella oneidensis* by dissipating electrons of the quinol pool. Sci. Rep..

[19] Meng Q, Yin J, Jin M, Gao H (2018). Distinct nitrite and nitric oxide physiologies in *Escherichia coli* and *Shewanella oneidensis*. Appl. Environ. Microbiol..

[20] Meng Q, Sun Y, Gao H (2018). Cytochromes *c* constitute a layer of protection against nitric oxide but not nitrite. Appl. Environ. Microbiol..

[21] Yoshikawa S, Shimada A (2015). Reaction mechanism of cytochrome *c* oxidase. Chem. Rev..

[22] Borisov VB, Gennis RB, Hemp J, Verkhovsky MI (2011). The cytochrome *bd* respiratory oxygen reductases. Biochim. Biophys. Acta.

[23] Le Laz S (2016). Expression of terminal oxidases under nutrient-starved conditions in *Shewanella oneidensis*: detection of the A-type cytochrome *c* oxidase. Sci. Rep..

[24] Yin J (2015). Regulation of nitrite resistance of the cytochrome *cbb*_3_ oxidase by cytochrome *c* ScyA in *Shewanella oneidensis*. Microbiol. Open.

[25] Marrs B, Gest H (1973). Genetic mutations affecting the respiratory electron-transport system of the photosynthetic bacterium *Rhodopseudomonas capsulata*. J. Bacteriol..

[26] Hammer ND, Schurig-Briccio LA, Gerdes SY, Gennis RB, Skaar EP (2016). CtaM is required for menaquinol oxidase *aa*_3_ function in *Staphylococcus aureus*. mBio.

[27] Andries K (2005). A diarylquinoline drug active on the ATP synthase of *Mycobacterium tuberculosis*. Science.

[28] Lamprecht DA (2016). Turning the respiratory flexibility of *Mycobacterium tuberculosis* against itself. Nat. Commun..

[29] Hunter CJ (2004). Inhaled nebulized nitrite is a hypoxia-sensitive NO-dependent selective pulmonary vasodilator. Nat. Med..

[30] May B, Young L, Moore AL (2017). Structural insights into the alternative oxidases: are all oxidases made equal?. Biochem. Soc. Trans..

[31] Allison KR, Brynildsen MP, Collins JJ (2011). Metabolite-enabled eradication of bacterial persisters by aminoglycosides. Nature.

[32] Meylan S (2017). Carbon sources tune antibiotic susceptibility in *Pseudomonas aeruginosa* via tricarboxylic acid cycle control. Cell Chem. Biol..

[33] Arai H (2014). Enzymatic characterization and *in vivo* function of five terminal oxidases in *Pseudomonas aeruginosa*. J. Bacteriol..

[34] Kawakami T, Kuroki M, Ishii M, Igarashi Y, Arai H (2010). Differential expression of multiple terminal oxidases for aerobic respiration in *Pseudomonas aeruginosa*. Environ. Microbiol..

[35] Osamura T, Kawakami T, Kido R, Ishii M, Arai H (2017). Specific expression and function of the A-type cytochrome *c* oxidase under starvation conditions in *Pseudomonas aeruginosa*. PLoS ONE.

[36] Brown TH, Alford RH (1984). Antagonism by chloramphenicol of broad-spectrum beta-lactam antibiotics against *Klebsiella pneumoniae*. Antimicrob. Agents Chemother..

[37] Ankomah P, Johnson PJT, Levin BR (2013). The pharmaco –, population and evolutionary dynamics of multi-drug therapy: experiments with *S. aureus* and *E. coli* and computer simulations. PLoS Pathog..

[38] Johansen HK, Jensen TG, Dessau RB, Lundgren B, Frimodt-Møller N (2000). Antagonism between penicillin and erythromycin against *Streptococcus pneumoniae in vitro* and *in vivo*. J. Antimicrob. Chemother..

[39] Rittershaus ESC, Baek S-H, Sassetti CM (2013). The normalcy of dormancy: common themes in microbial quiescence. Cell Host Microbe.

[40] Jiang Y (2014). Protection from oxidative stress relies mainly on derepression of OxyR-dependent KatB and Dps in *Shewanella oneidensis*. J. Bacteriol..

[41] Jin M (2013). Unique organizational and functional features of the cytochrome *c* maturation system in *Shewanella oneidensis*. PLoS ONE.

[42] Meng Q, Liang H, Gao H (2018). Roles of multiple KASIII homologues of *Shewanella oneidensis* in initiation of fatty acid synthesis and in cerulenin resistance. Biochim. Biophys. Acta.

[43] Chen H, Luo Q, Yin J, Gao T, Gao H (2015). Evidence for the requirement of CydX in function but not assembly of the cytochrome *bd* oxidase in *Shewanella oneidensis*. Biochim. Biophys. Acta.

[44] Fu H, Jin M, Ju L, Mao Y, Gao H (2014). Evidence for function overlapping of CymA and the cytochrome *bc*_1_ complex in the *Shewanella oneidensis* nitrate and nitrite respiration. Environ. Microbiol..

