# Supplementary material for: Nitrite modulates aminoglycoside tolerance by inhibiting cytochrome heme-copper oxidase in bacteria
Source: Commun Biol. 2020 May 27;3:269. doi: 10.1038/s42003-020-0991-4 (PMC7253457; doi:10.1038/s42003-020-0991-4)
Supplement: Supplementary file 1 — Supplemental Information [file 42003_2020_991_MOESM1_ESM.pdf]

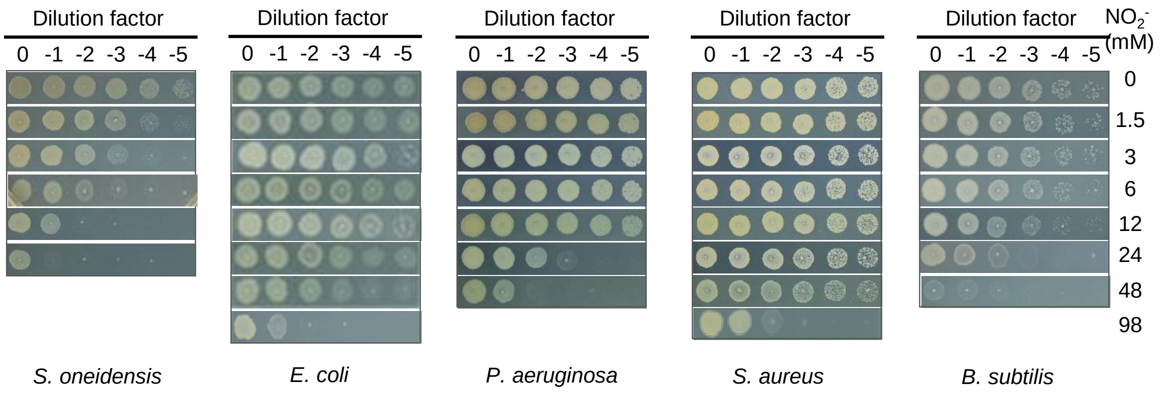

Supplementary figure 1 Nitrite susceptibility of *S. oneidensis, E. coli*, *P. aeruginosa, S. aureus,* and *B. subtilis*. The spot dilution assay of indicated bacteria prepared as in Fig. 1F. Nitrite was added as indicated. Photos were taken after incubation for 18 hours. Experiments were performed independently at least 5 times, and data were presented as values representative of similar results.

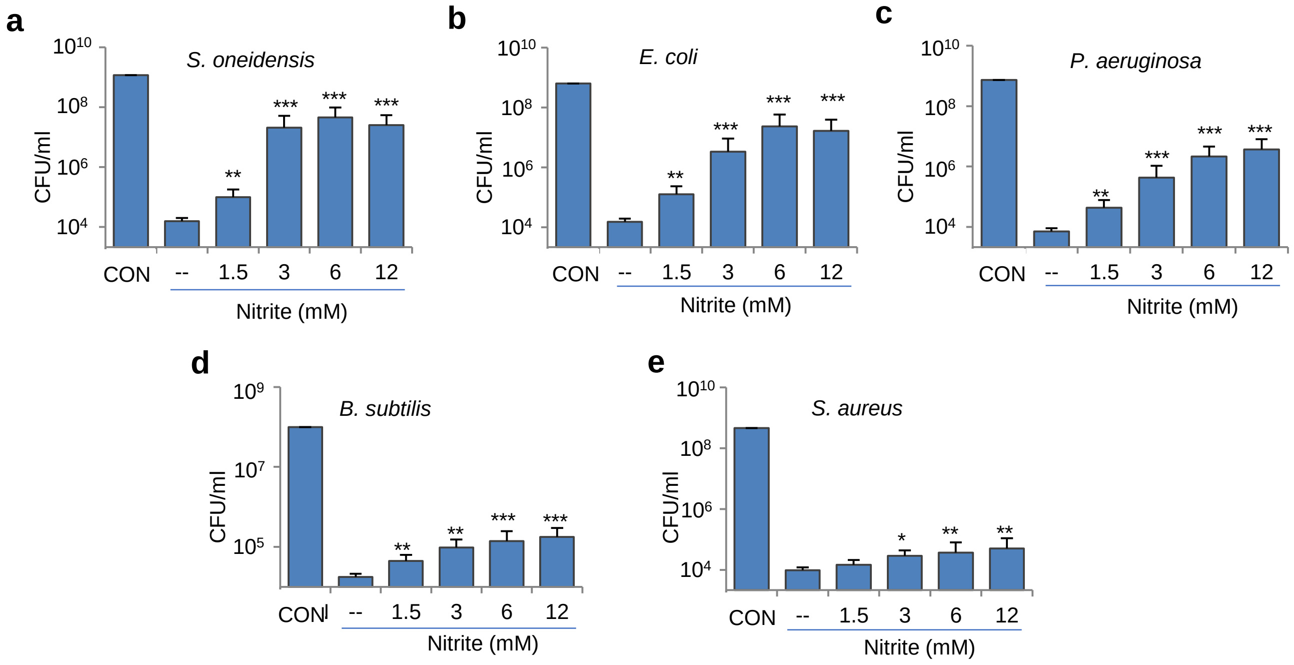

Supplementary figure 2 Nitrite modulates streptomycin (Str, 5X MIC) susceptibility of various bacteria. a-e Time-kill analysis of Gm (5X MIC) and nitrite combination for *S. oneidensis*, *E. coli*, *P. aeruginosa*, *B. subtilis*, and *S. aureus.* For all strains, cultures at the mid-log phase were used and their cell numbers before the treatment were given as the control (CON). Values shown were the number of viable cells 4 h after the treatment began. Combination therapy was compared against monotherapy with Gm, between which statistically significant difference caused by nitrite were given (*, $P < 0.05$; **, $P < 0.01$; ***, $P < 0.001$). Results were recorded after incubation of 18 h. Experiments were performed independently at least 3 times, and data were presented as mean ± SD.

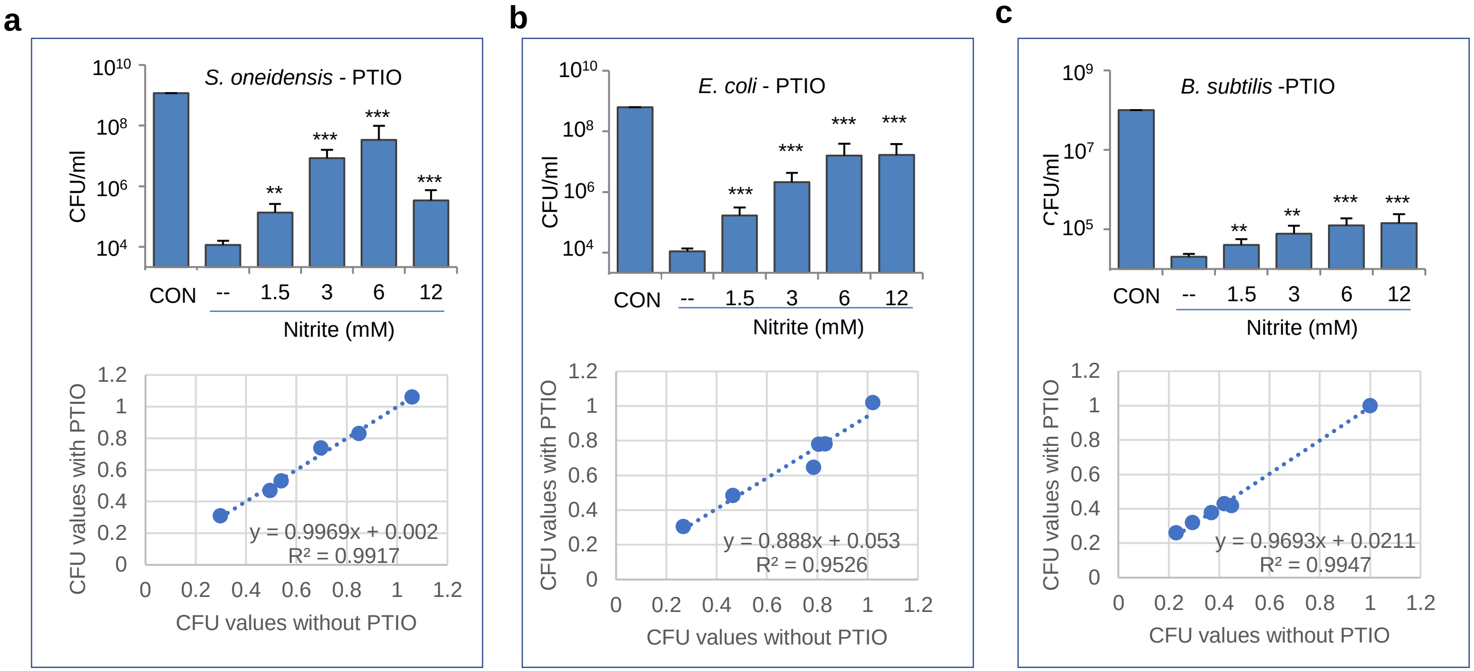

Supplementary figure 3 Nitrite but not NO modulates Gm (5X MIC) susceptibility of various bacteria. a-c Upper panel: time-kill analysis of Gm (5X MIC) and nitrite combination for *S. oneidensis*, *E. coli*, and *B. subtilis* in the presence of 2 mM NO-Scavenger PTIO, which is over 100 times more than needed to scavenge endogenous NO produced in *E. coli* and *B. subtilis,* according to the stoichiometric calculation. Combination therapy was compared against monotherapy with Gm, between which statistically significant difference caused by nitrite were given (*, $P < 0.05$; **, $P < 0.01$; ***, $P < 0.001$). Results were recorded after incubation of 18 h. Lower panel: statistic analysis of data without and with PTIO. Experiments were performed independently at least 3 times, and data were presented as mean ± SD.

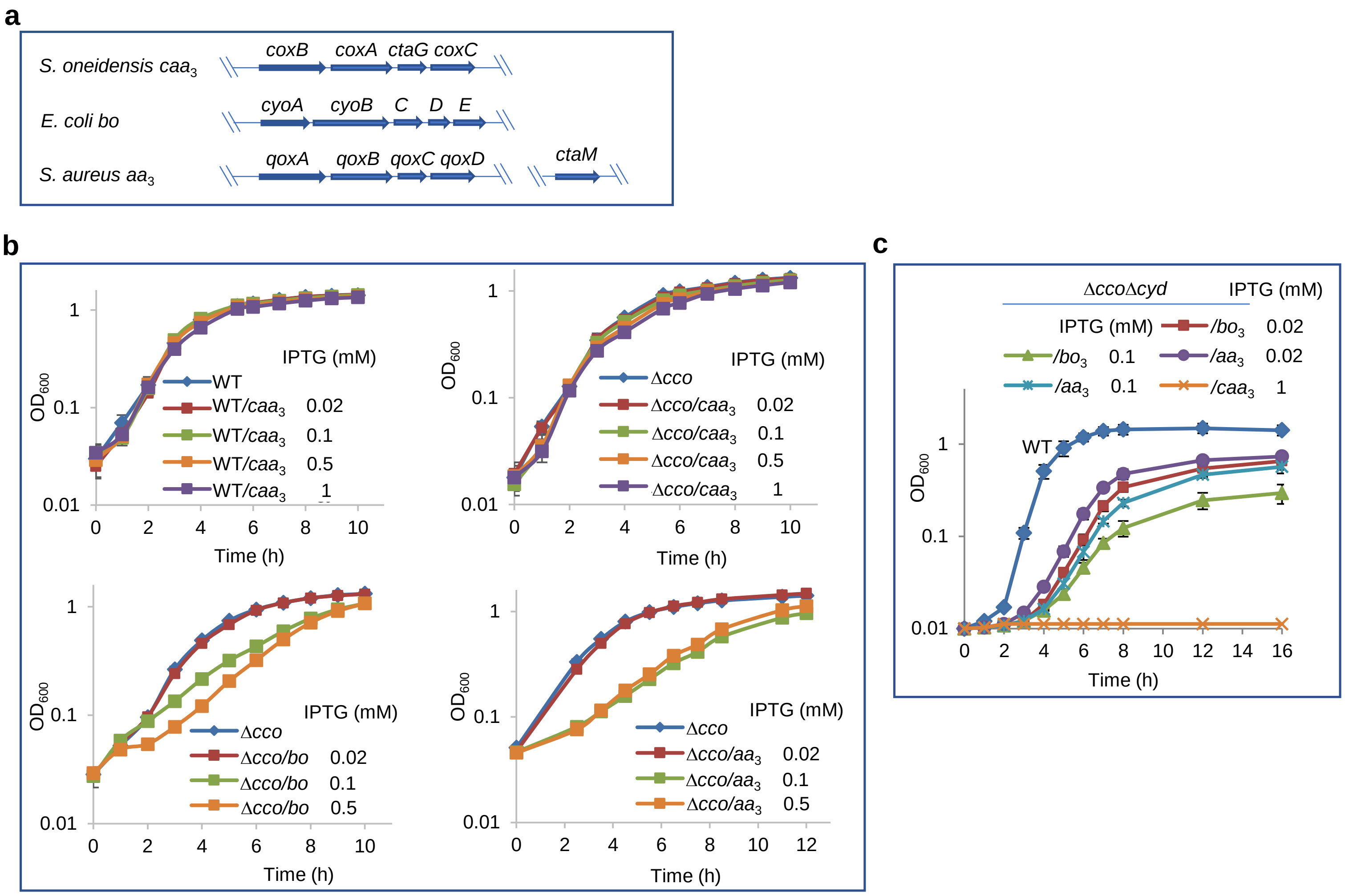

Supplementary figure 4 Expression of various oxidases and their effects on growth of *S. oneidensis*. a Genes shown in the chromosomes were cloned and placed after IPTG-inducible promoter P*tac* within pHGE-Ptac. **b** Growth of *S. oneidensis* WT and *cbb*$_3$-deficient strains in LB with increasing expression of *caa*$_3$, *bo*, or *aa*$_3$. **c** Growth of *S. oneidensis cbb*$_3$*-bd* -deficient strains in LB with expression of *caa*$_3$, *bo*, or *aa*$_3$ with IPTG at indicated concentrations. Data are shown as mean ± SD from at least three experiments.

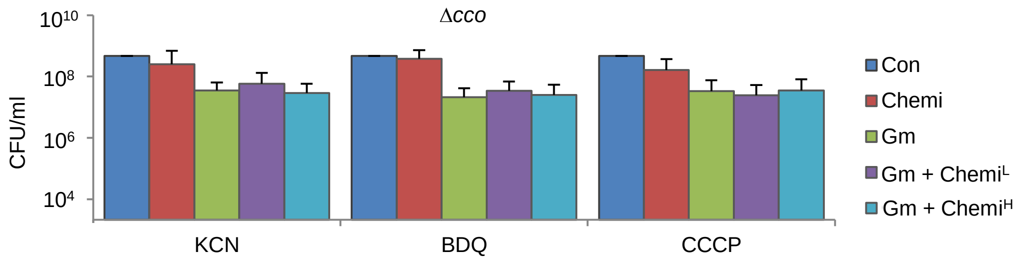

Supplementary figure 5 PMF but not respiration dictates uptake of aminoglycosides. Time-kill analysis of Gm (5X MIC) and KCN, BDQ, or CCCP combination for Δ*cco*. Chemi, one of the chemicals with superscript L and H representing low and high concentrations (100 and 500 μM for KCN, 1 and 5 μM for BDQ, 10 and 50 μM for CCCP). Experiments were performed independently at least 3 times, and data were presented as mean ± SD.

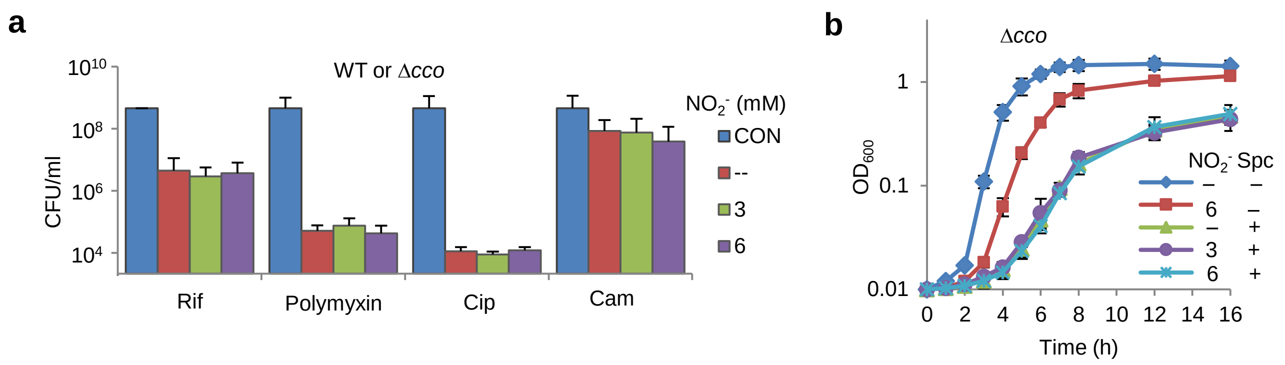

Supplementary figure 6 HCOs are dispensable in modulating susceptibility to antibiotics other than aminoglycosides. a Time-kill analysis of Rif, polymyxin, Cip, and Cam (5X MIC) and nitrite combination for WT and Δ*cco*. **b** Growth analysis of spectinomycin (Spc, 0.5X MIC) and nitrite combination for relevant *S. oneidensis* strains in liquid LB. Growth was recorded by measuring OD_{600} values of cultures. Experiments were performed independently at least 3 times, and data were presented as mean ± SD.
