# Supplementary material for: Nitrite modulates aminoglycoside tolerance by inhibiting cytochrome heme-copper oxidase in bacteria
Source: Commun Biol. 2020 May 27;3:269. doi: 10.1038/s42003-020-0991-4 (PMC7253457; doi:10.1038/s42003-020-0991-4)
Supplement: Supplementary file 2 — Description of Additional Supplementary Files [file 42003_2020_991_MOESM2_ESM.pdf]

Description of additional supplementary files

Supplementary Data 1:
Source data underlying plots shown in the main figures.
